# Biological Characteristics and Domestication of *Dichomitus squalens* and the Antioxidant Activity of Its Cultivated Fruiting Bodies

**DOI:** 10.3390/jof11080594

**Published:** 2025-08-15

**Authors:** Li-Bo Wang, Zheng-Xiang Qi, Tao Zhang, Ke-Qing Qian, Hai-Yan Lv, Bo Zhang, Yu Li

**Affiliations:** 1 Engineering Research Center of Chinese Ministry of Education for Edible and Medicinal Fungi, Jilin Agricultural University, Changchun 130118, China; wlblucky@126.com (L.-B.W.); qzx7007@126.com (Z.-X.Q.); 18844181185@163.com (T.Z.); 15670567112@163.com (K.-Q.Q.); 2 College of Mycology, Jilin Agricultural University, Changchun 130118, China; 3 College of Horticulture, Jilin Agricultural University, Changchun 130118, China; lvhaiyan@jlau.edu.cn; 4 Industrial Development Institute for Plants, Animals and Fungi Integration of Biyang County, Zhumadian 463799, China; 5 Inner Mongolia Innovation Center of Biological Breeding Technology, Hinggan League Institute of Agricultural and Husbandry Sciences, Ulanhot 137400, China

**Keywords:** *Dichomitus squalens*, orthogonal experiments, liquid strains, hydroxyl radical, superoxide anion radical

## Abstract

Single-factor and orthogonal experiments were conducted to investigate the biological characteristics of *Dichomitus squalens* strains isolated from wild fruiting bodies collected in Tekes County, Xinjiang Uygur Autonomous Region. Building upon the optimal mycelial culture conditions identified, domestication cultivation studies were performed, including experiments to induce fruiting body formation. Liquid strains were inoculated into substrates to monitor developmental stages from primordia formation to mature fruiting bodies, with macroscopic characteristics recorded throughout the cultivation process. Crude polysaccharides were extracted from the cultivated fruiting bodies using the water extraction and ethanol precipitation method. The scavenging rates of these polysaccharides against hydroxyl radicals (OH^−^) and superoxide anion radicals (O_2_^−^) were measured to evaluate their in vitro antioxidant activity. Results demonstrated that the optimal growth conditions for *D. squalens* were as follows: sucrose as the preferred carbon source, yeast extract powder as the optimal nitrogen source, a pH of 5.0, and a temperature of 30 °C. Among these factors, pH exerted the most significant influence on the mycelial growth rate, followed by nitrogen source, carbon source, and temperature. Mature fruiting bodies developed approximately 57 days after inoculation with liquid strains. The crude polysaccharide extraction yield from the cultivated fruiting bodies reached 7.07%, with a total polysaccharide content of 24.69% in the extract. The crude polysaccharides exhibited potent radical scavenging activity: at a concentration of 5.0 mg/mL, the hydroxyl radical scavenging rate was 56.74%, while the superoxide anion radical scavenging rate reached 78.3%. These findings indicate that *D. squalens* possesses significant antioxidant potential.

## 1. Introduction

*Dichomitus squalens* (P. Karst.) D.A. Reid is classified within the Basidiomycota, Agaricomycetes, Polyporales, Polyporaceae, and *Dichomitus*. This species exhibits a broad distribution across Northern Europe, North America, and Asia. In China, it is predominantly found in Northeast China, North China, Northwest China, Tibet, and Central China, where it frequently colonizes coniferous trees [1].

*Dichomitus squalens*, an effective white-rot fungus, holds substantial promise for the breakdown of lignocellulosic substrates and plant biomass. Ligninolytic enzymes, such as laccase (Lac), manganese peroxidase (MnP), and lignin peroxidase (LiP), play a crucial role in the degradation and conversion of plant biomass by breaking down the primary components of plant cell walls [2]. *D. squalens* exhibits significant lignocellulose-degrading capabilities for both hardwood and softwood, demonstrating particularly high efficiency in lignin depolymerization [3]. In competitive interactions with other white-rot fungi, its increased ligninolytic activity enhances its ecological adaptability and survival fitness [4].

*Dichomitus squalens* demonstrates remarkable efficacy in the degradation of environmental organic pollutants. Šušla et al.demonstrated that when immobilized in a fixed-bed reactor, *D. squalens* effectively degrades the recalcitrant dyes Remazol Brilliant Blue R and Reactive Orange 16 [5]. The decolorization mechanism is closely associated with laccase activity.

In recent years, *Dichomitus squalens* has gained recognition as a novel reference species among white-rot fungi, with significant advancements in its genomic and transcriptomic research. Concerning the role of specific enzymes in plant cell wall degradation, Daly et al. elucidated the complexity of the regulatory mechanisms employed by *D. squalens* for the utilization of plant biomass utilization under varying carbohydrate availability [6]. Under conditions of elevated glucose concentrations, approximately 7% of gene expression was inhibited, primarily affecting genes associated with carbohydrate-active enzymes (CAZymes) and carbon metabolism related to plant biomass utilization. This finding indicates that *D. squalens* can dynamically modulate its gene expression in natural environments to adapt to various nutritional conditions, thereby enhancing its efficiency in biomass degradation. Subsequent research conducted by Li et al. has demonstrated that the genome of *D. squalens* contains numerous genes encoding enzymes associated with the degradation of plant biomass [7]. Notably, these include carbohydrate-active enzymes (CAZymes), which are proficient in breaking down lignocellulose and other complex plant polymers.

With the rapid advancement of genome editing technologies, the CRISPR/Cas9 system has achieved groundbreaking progress in fungal genetic engineering. Kowalczyk et al. advanced the field of precise genome editing in *Dichomitus squalens* by developing a dual homologous recombination template vector system [8]. This system successfully illustrated the capability for targeted gene knockout and allele replacement in filamentous fungi. Daly et al. established a standardized protoplast preparation protocol based on a commercial enzymatic digestion system [9], where the application of a novel composite enzymatic cocktail significantly enhanced the transformation efficiency of *D. squalens*, thereby laying a methodological foundation for subsequent genetic manipulations. Current research on *D. squalens* primarily emphasizes its efficacy in wood degradation [10]. However, the regulatory mechanisms by which environmental factors influence its mycelial development, secondary metabolite synthesis, and other biological characteristics have yet to be systematically elucidated.

In recent years, fungal polysaccharides have become a central focus in biomedical research. Fungal polysaccharides, as essential bioactive constituents of both edible and medicinal fungi, have attracted significant scholarly interest due to their physiological properties. These include their ability to scavenge or reduce the generation of free radicals, their antioxidant activity, and their low toxicity with minimal side effects [11]. Under normal physiological conditions, the production and elimination of free radicals in organisms maintain a dynamic equilibrium. Nevertheless, an excessive accumulation of free radicals can result in damage to cellular components and activate specific signaling pathways, thereby accelerating the aging process and precipitating the onset of diseases [12]. Conversely, comprehensive studies on polysaccharides extracted from *D. squalens* are limited.

To address existing research gaps, this study first conducted a precise taxonomic identification of *Dichomitus squalens* and augmented this with an examination of its characteristics. Single-factor experiments were conducted to examine the impact of key ecological factors on hyphal growth, while orthogonal experiments were employed to analyze the interactive effects of environmental variables. Subsequent experiments on domestication cultivation were conducted to offer theoretical support for the artificial cultivation of *D. squalens*. Furthermore, this study conducted a preliminary investigation into the antioxidant activity of polysaccharides derived from *D. squalens*, thereby identifying a novel source of natural antioxidants and establishing a foundational material for the development of new medicinal fungal resources.

## 2. Materials and Methods

### 2.1. Experimental Strains and Specimens

The specimens of wild fruiting bodies employed in this study were sourced from Tekes County, Yili Kazakh Autonomous Prefecture, Xinjiang Uygur Autonomous Region. Wild strains were acquired through the tissue isolation method as described by [13] and were subsequently subjected to laboratory-based isolation and purification procedures. The specimens have been deposited in the Fungal Herbarium of Jilin Agricultural University (FJAU), collection numbers: FJAU66625–FJAU6628.

### 2.2. Formulations of Experimental Media

The solid PDA medium comprises peeled potatoes at a concentration of 200 g/L, glucose at 20 g/L, and agar at 20 g/L, with the pH remaining unadjusted.

The medium for the single-factor experiment on carbon sources comprised 20 g/L of the tested carbon source, 2 g/L of yeast extract, 2 g/L of peptone, 3 g/L of potassium dihydrogen phosphate, 1.5 g/L of magnesium sulfate, and 20 g/L of agar, with the pH left unadjusted. The control group medium was prepared without the inclusion of the tested carbon source.

The medium for the single-factor experiment on nitrogen sources comprised 20 g/L of the tested nitrogen source, 20 g/L of glucose, 3 g/L of potassium dihydrogen phosphate, 1.5 g/L of magnesium sulfate, and 20 g/L of agar, with the pH left unadjusted. The control group medium was identical, except for the omission of the tested nitrogen source.

The medium for the pH single-factor experiment consists of peeled potatoes at a concentration of 200 g/L, glucose at 20 g/L, agar at 20 g/L, and a 1 mol/L solution of either HCl or NaOH.

The medium for the temperature single-factor experiment was prepared in the same manner as the solid PDA medium formulation.

The orthogonal experiment medium comprises a carbon source at a concentration of 20 g/L, a nitrogen source at 20 g/L, potassium dihydrogen phosphate at 3 g/L, magnesium sulfate at 1.5 g/L, and agar at 20 g/L. Additionally, 1 mol/L HCl and NaOH solutions are utilized.

The liquid medium comprises glucose at a concentration of 20 g/L, yeast extract powder at 5 g/L, potassium dihydrogen phosphate at 2 g/L, and magnesium sulfate at 1 g/L, with the pH remaining unadjusted.

The composition of the third-stage spawn substrate is as follows: coarse sawdust constitutes 62%, fine sawdust 16%, wheat bran 18%, soybean meal 2%, lime 0.5–1%, and gypsum 1%.

### 2.3. Species Identification

This study employed both wild and cultivated fruiting bodies as subjects of investigation. Species identification was achieved through macroscopic and microscopic morphological observations [1,14], and molecular biological techniques were utilized to confirm the sequencing results for both domesticated and wild fruiting bodies.

Macroscopic morphological characteristics and habitat information were systematically recorded. A microscopic analysis of basidiospores, cystidia, basidia, basidioles, vegetative hyphae, and generative hyphae was conducted utilizing 5% KOH and 1% Congo red as mounting media.

DNA was extracted from both wild and domesticated fruiting bodies utilizing a novel plant genomic DNA extraction kit (Jiangsu Kangwei Century Biotechnology Co., Ltd., Taizhou, China). Subsequent PCR amplification and sequencing were conducted using ITS1 and ITS4 primers [15]. The primer fragments ITS1 and ITS4 were utilized with the following sequences (5′→3′): ITS1, TCCGTAGGTGAACCTGCGG and ITS4, TCCTCCGCTTATTGATATGC. The polymerase chain reaction (PCR) amplification protocol is as follows, with a total reaction volume of 25 µL: sterile water, 14.5 µL; 5× PCR buffer, 5 µL; dNTPs, 1 µL; forward and reverse primers, 1 µL each; DNA template, 2 µL; and Taq DNA polymerase, 0.5 µL. The amplification conditions are specified as follows: initial denaturation at 95 °C for 3 min; cycling (34 cycles) includes denaturation at 94 °C for 40 s, annealing at 55 °C for 45 s, and extension at 72 °C for 1.5 min; final extension at 72 °C for 10 min; and a hold at 4 °C.

Phylogenetic analyses were performed utilizing Maximum Likelihood (ML) and Bayesian Inference (BI) methodologies, with multigene phylogenetic trees constructed using MEGA 11.0 and PhyloSuite 1.2.2. These analyses corroborated the taxonomic classification of the specimens under investigation.

### 2.4. Biological Characterization Studies

Strain activation was conducted on PDA medium. Upon the expansion of the mycelial to the periphery of a 90 mm Petri dish, hyphal samples were extracted using an aseptic cork borer (Φ = 6 mm) and subsequently transferred to the central region of experimental media under sterile conditions for cultivation.

Subsequent to inoculation, a constant-temperature cultivation system was implemented in accordance with the experimental groupings. Observations began with the initiation of hyphal growth, and the morphological characteristics of the colonies were documented every 24 h. The diameters of the colonies were measured and recorded utilizing the cross-diameter method [16]. Observations were concluded when any experimental group reached the periphery of the Petri dish. The linear hyphal growth rate, expressed in millimeters per day (mm/d), was determined using the formula v = (D − d)/t, where D denotes the final diameter, d the initial diameter, and t the duration of cultivation. Concurrently, photographic documentation of the colony morphology was conducted.

#### 2.4.1. Single-Factor Experiment

(1) Carbon source single-factor experimental design

Five treatment groups were established, each utilizing a different monosaccharide carbon source: glucose, sucrose, soluble starch, dextrin, and maltose. Peptone and yeast extract were used fixed nitrogen sources for medium preparation. Additionally, a control group with no carbon source added was included [17]. Following inoculation, all media were maintained at a temperature of 25 °C in a constant-temperature incubator under dark conditions.

(2) Nitrogen source single-factor experimental design

Five treatment groups were established using monosaccharide nitrogen sources, specifically peptone, yeast extract powder, ammonium chloride, diammonium phosphate, and urea, with glucose serving as the fixed carbon source for medium preparation. A parallel control group, which contained no added nitrogen, was also included [17]. Following inoculation, all media were maintained at a constant temperature of 25 °C in an incubator under dark conditions.

(3) pH gradient experimental design

pH gradients ranging from 5.0 to 9.0, with an increment of ΔpH = 1.0, were established in sterilized PDA solutions by adjusting with 1 mol/L HCl and NaOH solutions [18]. Following inoculation, the media were maintained at a temperature of 25 °C in a constant-temperature incubator under dark conditions.

(4) Temperature gradient experimental design

Following the inoculation of PDA medium with the test strains, a series of temperature gradients (15–35 °C, ΔT = 5 °C) was established. The samples were then incubated in constant-temperature incubators under dark conditions, as described by Zhang et al. [19].

Each single-factor experiment comprised five replicates for each treatment group. The data on hyphal growth rates were analyzed using Excel 2024. Data on hyphal growth rates were imported into SPSS version 29 for the purpose of conducting a one-way ANOVA. Assuming homogeneity of variance, post hoc tests were selected as “LSD” and “Duncan”. The analyses were performed at significance levels of 0.05 and 0.01, respectively, with the “Descriptives” and “Homogeneity of variance test” options enabled for both analyses.

#### 2.4.2. Orthogonal Experiments

An L9(3^4^) orthogonal matrix was constructed based on optimal three-level selections for each factor, as derived from the results of single-factor experiments. The experimental media were prepared in accordance with the orthogonal array design, with each treatment group consisting of five replicates. The hyphal growth rate data were subjected to statistical analysis using Excel 2024, while range analysis and ANOVA were conducted utilizing SPSS 29 software [19]. Orthogonal experiment variance analysis: to analyze the mycelial growth rate, input the data into SPSS 29 software. Select “Univariate” under the “General Linear Model,” configure the main effects model, incorporate various experimental treatment groups, and conduct the between-subject effects test analysis. Orthogonal Experiment Range Analysis (Intuitive Analysis): access the built-in range analysis panel in SPSSAU (https://spssau.com/indexs.html November 2024), input the mycelial growth rate data, and complete the analysis.

### 2.5. Domestication Cultivation Experiments

#### 2.5.1. Liquid Strain Preparation

The liquid medium was distributed into 500 mL conical flasks, with each flask containing 200 mL. The media were then sterilized using an autoclave at 121 °C for 30 min. After sterilization, the media were placed in a laminar flow cabinet to cool until they reached ambient temperature (25 ± 1 °C). Three to four mycelial plugs were aseptically transferred into each flask. The flasks were subsequently sealed and incubated in a constant-temperature shaker set at 28 °C and 150 rpm to facilitate submerged cultivation [20].

#### 2.5.2. Production and Mycelial Colonization Management of Cultivation Spawn

Coarse sawdust, fine sawdust, and wheat bran were mixed in specific proportions and subjected to quantitative hydration. The moisture content of the substrate was calibrated to 60 ± 2% using a humidity sensor, followed by a 12 h equilibration period to ensure uniform water absorption. Soybean meal, lime, and gypsum were subsequently integrated into the moist substrate in accordance with specified ratios. The blended substrate was allocated into cultivation bags (800 g per bag) and subjected to sterilization through autoclaving at 121 °C for 30 min. After sterilization, the bags were placed in a laminar flow cabinet for ambient cooling over a period of 12 h. Each bag was inoculated with 20 mL of liquid strain and subsequently incubated at 25 °C in constant-temperature incubators under dark conditions to facilitate the development of the fruiting body [21].

#### 2.5.3. Primordia Induction and Fruiting Body Development

After the cultivation bags were fully colonized by dense and robust mycelia, a subsequent post-maturation phase lasting seven days was implemented. Following the removal of the closures, the bags were transferred to artificial climate chambers. The environmental conditions within these chambers were maintained at a temperature range of 18–20 °C and a humidity level of 90% to promote the development of primordia. Three to four days post-primordiation, the bags were resealed, and V-shaped incisions were created adjacent to the areas containing the primordia. The colonized bags were then relocated to climate-controlled chambers, which were maintained at a temperature of 24–25 °C and a humidity level of 90% to promote the development of fruiting bodies [22].

### 2.6. Crude Polysaccharide Extraction

Crude polysaccharides were extracted from both wild and cultivated fruiting bodies of *Dichomitus squalens* using the water extraction and ethanol precipitation method [23]. The fruiting bodies were desiccated to a constant weight, subsequently pulverized into a fine powder using liquid nitrogen in a mortar, and then combined with double-distilled water at a solid-to-liquid ratio of 1:20. The mixture underwent hot water extraction at 90 °C for a duration of 3 h in a water bath. The crude extract was subjected to vacuum filtration to yield an aqueous solution, which was subsequently concentrated to one-quarter of its original volume utilizing a rotary evaporator. The concentrated aqueous solution was mixed with absolute ethanol in a volume ratio of 1:4, resulting in a final alcohol concentration of 80%. The mixture was then stored at 4 °C for 12 h to facilitate ethanol precipitation.

### 2.7. Total Polysaccharide Content Determination

The polysaccharide content of *Dichomitus squalens* was determined using the phenol-sulfuric acid method [24]: an eight-point glucose standard curve was established, ranging from 0 to 100 μg/mL (0, 10, 20, 30, 40, 60, 80, and 100 μg/mL). Concurrently, a crude polysaccharide sample solution with a concentration of 100 μg/mL was prepared. Both the sample and standard solutions were sequentially combined with a 6% phenol solution and concentrated sulfuric acid in a volumetric ratio of 2:1:5 (sample/phenol/H_2_SO_4_). Following the completion of the reaction, the absorbance was recorded at 490 nm utilizing a microplate reader. The glucose standard curve was constructed using Excel, and the absorbance values of the samples were interpolated to determine the total polysaccharide content.

### 2.8. Antioxidant Activity Determination

Solutions of crude polysaccharide samples were prepared at varying concentrations of 0.5, 1, 1.5, 2, 3, 4, and 5 mg/mL. The hydroxyl radical scavenging capacity and superoxide anion radical scavenging capacity were evaluated utilizing commercial assay kits (Suzhou Keming Biotechnology Co., Ltd., Suzhou, China), with three independent experimental replicates conducted. Mean values were computed, and standard curves were constructed to quantify antioxidant activity.

## 3. Results and Analysis

### 3.1. Species Identification

*Dichomitus squalens* (P. Karst.) D.A. Reid, Revta Biol., Lisb. 5 (1–2): 150 (1965)

Wild fruiting bodies were annual to biennial, imbricate, and suberose in texture. The pileus was niveous and semiorbicular, measuring 17–52 × 16–27 mm, laevis to faintly spinosus, devoid of zonatus, and transitioning to lacteus upon maturation and desiccation. The margin exhibited sinuatus morphology with slight involutus. The context (flesh) was niveous, attaining a thickness of 3–10 mm, demonstrating fibrillose growth patterns. The tubes were niveous, extending 2–6 mm in length, with rhombic to orbicular pores (3–5 pores per millimeter), concolorous with the tubes, presenting a lacteus appearance when desiccated.

**Figure 1:**
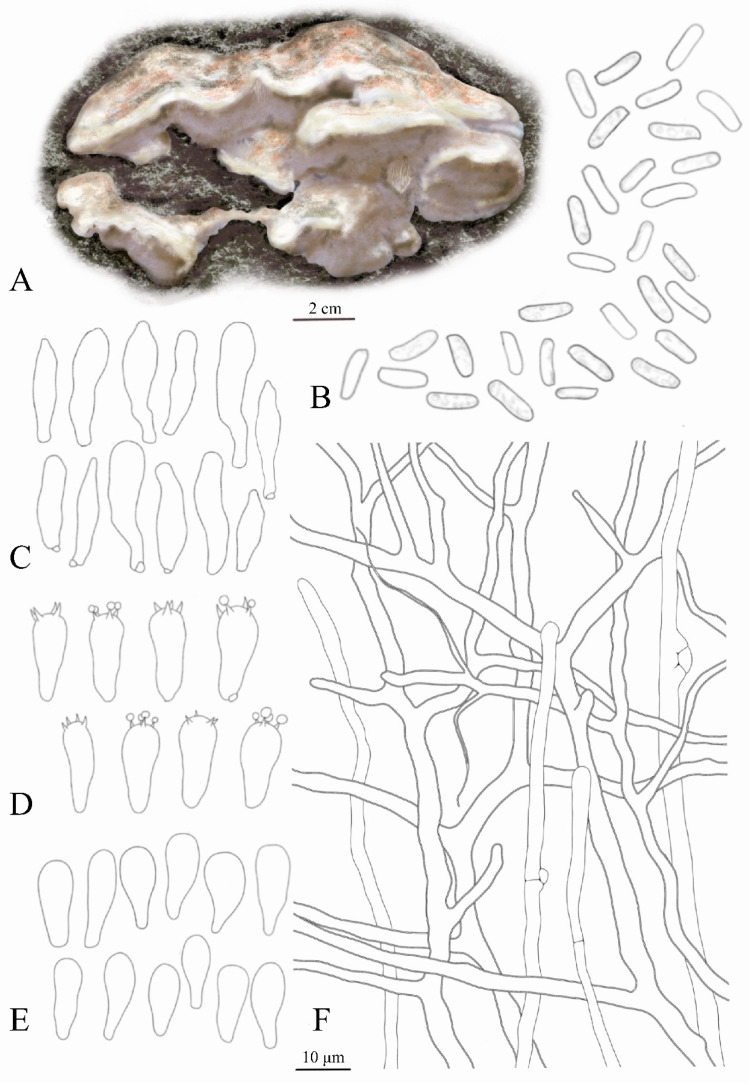
*Dichomitus squalens*: (**A**) Macroscopic characteristics. (**B**) Basidiospores. (**C**) Cystidia. (**D**) Basidia. (**E**) Basidioles. (**F**) Vegetative hyphae and Generative hyphae. Scale bar: 2 cm (**A**), 10 μm (**B**–**F**).

Basidiospores (8.2)8.5–10.5(11) × (3)3.1–3.7(5.2) μm, cylindrical, hyaline, thin-walled, smooth, occasionally containing guttules. Basidia (13)14.5–17.2(20) × (5.7)6–8(8.3) μm, clavate, hyaline, thin-walled, bearing four sterigmata. Basidioles (12)14–16.5(18) × (5.7)6–7.7(8) μm, similar in shape and size to basidia. Cystidia (15)17–23(27) × (4.5)4–6(6.5) μm, thin-walled, hyaline, irregularly elongate-clavate to fusiform with slightly protruding apices. Vegetative hyphae 1.5–4.5 μm, dichotomously branched, hyaline, main trunks 3–4.5 μm thick-walled, tapering terminally into flagelliform entangled hyphae. Generative hyphae 1.4–4 μm, thin-walled, hyaline, with distinct clamp connections (Figure 1). These characteristics align with descriptions of *Dichomitus squalens* by Li et al. [1], Zhao [25], and Jancovičová [26].

Habitat: Solitary on decayed wood in *Picea schrenkiana* forests.

Distribution: This species is predominantly located in Northern Europe, North America, and Asia. In China, its presence is primarily noted in Northeast China, North China, Northwest China, Tibet, and Central China [1].

Studied specimens: China: Xinjiang: Tekes County, Xinjiang Uygur Autonomous Region, 25 July 2023, Zhengxiang Qi, FJAU66625; China: Xinjiang: Tekes County, Xinjiang Uygur Autonomous Region, 29 July 2023, Zhengxiang Qi, FJAU66626; China: Xinjiang: Tekes County, Xinjiang Uygur Autonomous Region, 15 August 2023, Zhengxiang Qi, FJAU66627.

**Figure 2:**
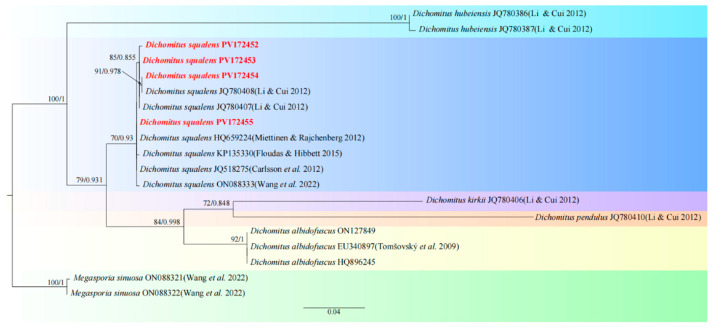
Phylogenetic tree of the *Dichomitus.* The best tree from the ML and Bl analysis of the nrITS + TEF1-a dataset. The two values of internal nodes, respectively, represent the maximum likelihood bootstrap (MLBP)/Bayesian posterior probability (BIPP). This study species is in bold and red font [27,28,29,30,31,32].

Molecular biological validation: Based on ITS sequence BLASTn analysis, the obtained sequence (PV172453) exhibited 100% query coverage and 100% nucleotide identity with the reference sequence of *Dichomitus squalens* (AM988622) (Table 1). The topological analysis demonstrated that the target sequence constituted a well-supported monophyletic clade (ML/BI support values = 70/0.93) alongside the *D. squalens* reference sequences JQ780408 and JQ780407, thereby conclusively affirming the taxonomic classification of the specimens under investigation (Figure 2).

The specimens and strains were conclusively identified as *Dichomitus squalens* through a combination of morphological and molecular biological evidence.

### 3.2. Biological Characterization Studies

#### 3.2.1. Single-Factor Experiments

**Figure 3:**
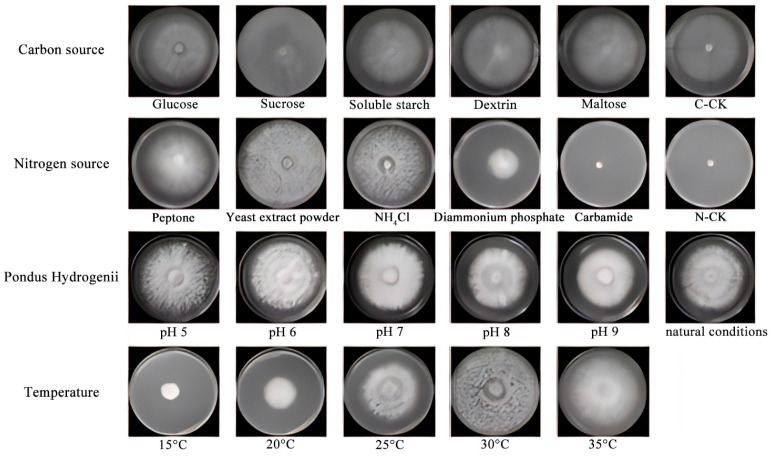
Mycelial growth vigor of different single factors of different treatments of *Dichomitus squalens*.

**Figure 4:**
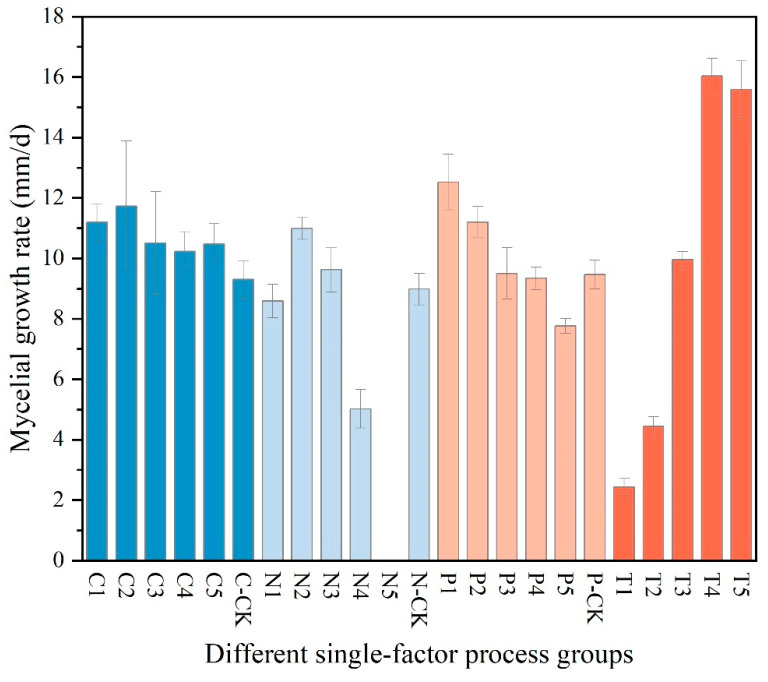
Histogram of the error of different single factors on the mycelial growth rate of *Dichomitus squalens*. The ‘C1’ in the image corresponds to the ‘C1’ horizontal treatment group in Table 2, and so on.

(1) The results of the single-factor experiment on carbon sources, as presented in Table 2, indicate that sucrose is the most effective carbon source for promoting the hyphal growth of *Dichomitus squalens*, achieving a growth rate of 11.73 ± 2.16 mm/d. Glucose was ranked second with a rate of 11.20 ± 0.61 mm/d, followed in descending order by soluble starch at 10.50 ± 1.70 mm/d, maltose at 10.48 ± 0.67 mm/d, and dextrin at 10.22 ± 0.66 mm/d. Under all experimental conditions utilizing a single carbon source, hyphal growth was relatively sparse (Figure 3 and Figure 4).
(2) The results of the nitrogen source single-factor experiment, as presented in Table 2, demonstrate that yeast extract powder is the most effective nitrogen source for promoting the hyphal growth of *Dichomitus squalens*, achieving a growth rate of 10.99 ± 0.36 mm/d. Ammonium chloride exhibited the second highest rate of growth at 9.63 ± 0.74 mm/d, followed sequentially by peptone at 8.59 ± 0.56 mm/d and diammonium phosphate at 5.02 ± 0.64 mm/d. Notably, the urea treatment group completely inhibited hyphal growth, resulting in 0 mm/d, whereas the nitrogen-free control group sustained basal metabolic activity at 8.99 ± 0.52 mm/d (Figure 3 and Figure 4).
(3) The results of the pH single-factor experiment, as presented in Table 2, indicate that pH exerts a significant influence on the hyphal growth of *Dichomitus squalens*. The optimal pH was 5.0, with a growth rate of 12.52 ± 0.92 mm/d, showing no significant difference from the pH 6.0 group (11.20 ± 0.52 mm/d). Under conditions ranging from neutral to alkaline pH levels (7.0–9.0), the growth rates demonstrated a linear decline (Figure 3 and Figure 4).
(4) The results of the temperature single-factor experiment, as presented in Table 2, indicate a significant dependence of *Dichomitus squalens* hyphal growth on temperature. The optimal temperature for growth was identified as 30 °C, with a growth rate of 16.03 ± 0.59 mm/d, which did not differ significantly from the growth rate at 35 °C (15.59 ± 0.95 mm/d). However, the growth rate at 30 °C was significantly higher than those observed at 25 °C (9.96 ± 0.27 mm/d), 20 °C (4.43 ± 0.33 mm/d), and 15 °C (2.44 ± 0.28 mm/d). Hyphal growth was robust and dense across all tested temperatures, as illustrated in Figure 3. Furthermore, within the temperature range of 15–30 °C, there was a significantly positive correlation between growth rates and temperature, as depicted in Figure 4.

#### 3.2.2. Orthogonal Experiments

**Figure 5:**
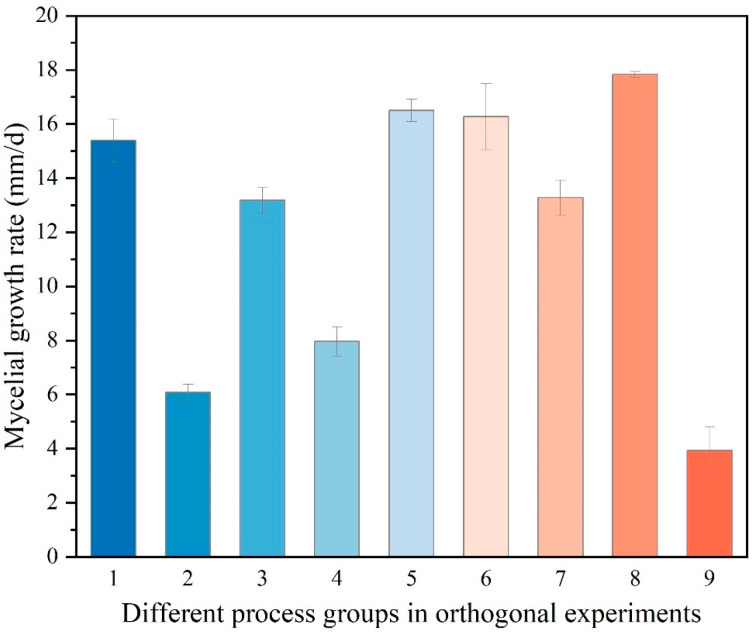
Mycelial growth rate of *Dichomitus squalens* in different process groups in orthogonal experiments. The ‘1’ treatment group in the image corresponds to ‘Text No. 1’ in Table 3, and so on.

**Figure 6:**
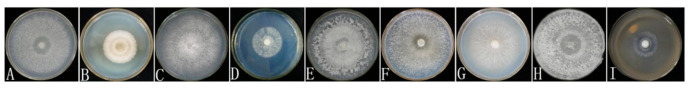
Mycelial growth vigor of different treatments of *Dichomitus squalens* in orthogonal experiments. (**A**) Processing 1, (**B**) processing 2, (**C**) processing 3, (**D**) processing 4, (**E**) processing 5, (**F**) processing 6, (**G**) processing 7, (**H**) processing 8, and (**I**) processing 9. “Processing 1” is equivalent to “Text No. 1” as presented in Table 3, and so on.

Analysis of the orthogonal experiments, as presented in Table 3, identified pH as the primary determinant of the hyphal growth rate of *Dichomitus squalens*. This was evidenced by the highest range value (R = 10.51), which significantly surpassed those of the nitrogen source (R = 2.34), carbon source (R = 2.03), and temperature (R = 1.11). The analysis of the mean value of K (K avg) determined the optimal parameter combination as follows: sucrose (carbon source level 2, K avg2 = 13.58 mm/d), yeast extract powder (nitrogen source level 2, K avg2 = 13.47 mm/d), pH 5.0 (pH level 1, K avg2 = 16.5 mm/d), and a temperature of 30 °C (K avg2 = 12.99 mm/d). The results demonstrated a high degree of consistency with the conclusions derived from single-factor experiments (Figure 5 and Figure 6). Notably, the absence of a significant difference between 30 °C and 35 °C in the single-factor temperature experiments suggests the possibility of experimental errors.

ANOVA (Table 4) demonstrated statistically significant effects in the following order: pH {F (2, 36) = 1018.38, *p* < 0.001} > nitrogen source {F (2, 36) = 45.60, *p* < 0.001} > carbon source {F (2, 36) = 42.64, *p* < 0.001) > temperature {F (2, 36) = 12.88, *p* < 0.001}, which is consistent with the findings from the range analysis.

### 3.3. Domestication Cultivation Trials

**Figure 7:**
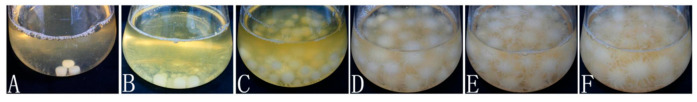
Hyphal growth activity of *Dichomitus squalens* liquid strains under varying cultivation durations. (**A**) 1 day. (**B**) 3 day. (**C**) 6 day. (**D**) 9 day. (**E**) 12 day. (**F**) 18 day.

Liquid strain cultivation: By the sixth day following inoculation, spherical mycelial aggregates, measuring 4–8 mm in diameter, with radially extending hyphae, were observed to form in the liquid medium (Figure 7C). By the twelfth day, these structures developed into echinate mycelial masses, measuring 10–12 mm in diameter, and exhibited a pure white coloration. They were uniformly distributed within the clarified liquid medium, indicating the successful preparation of the liquid strain (Figure 7E).

Solid-State Mycelium Cultivation: Each cultivation bag, containing 800 g of wet substrate, was inoculated with 20 mL of liquid strain and subsequently incubated in constant-temperature chambers under dark conditions, maintained at 25 ± 0.5 °C with a relative humidity of 65 ± 3%. Within approximately 30 days, the bags were completely enveloped by dense and robust mycelial colonization, signifying the completion of the vegetative growth phase.

**Figure 8:**
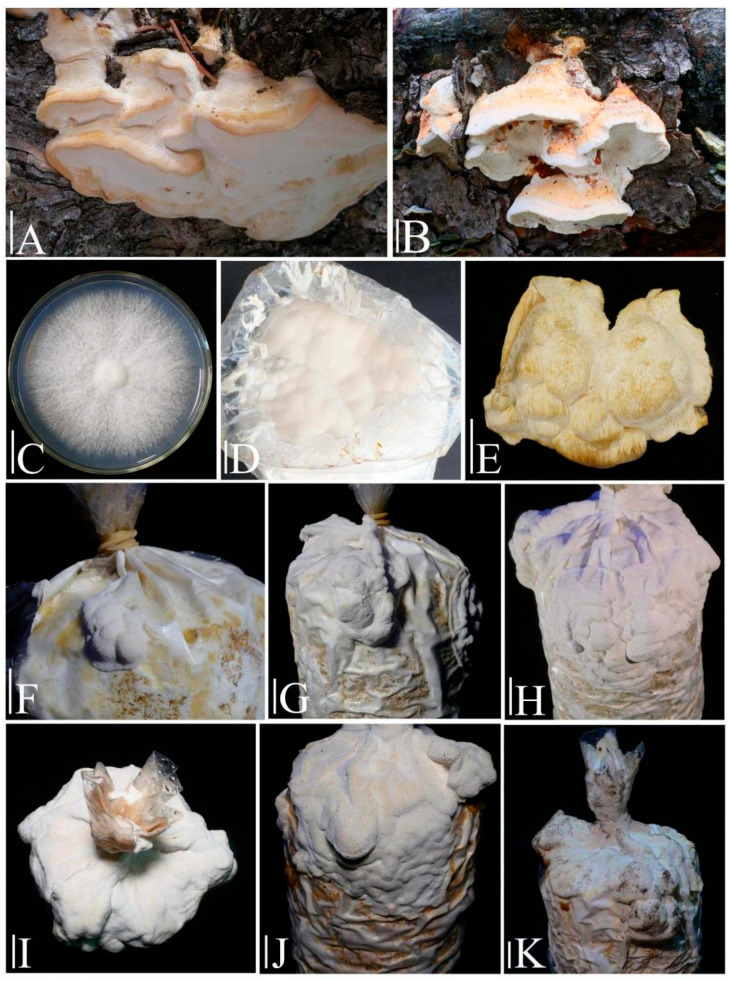
*Dichomitus squalens*. (**A**,**B**) Basidiocarps in situ. (**C**) Isolated mycelia. (**D**) Primoridia (**E**) Mature fruiting bodies grown. (**F**,**G**) Juvenile fruiting bodies. (**H**–**J**) Unmature fruiting bodies. (**K**) Mature fruiting bodies. Scale bars: 2 cm.

Primordia induction and fruiting body development: The bags containing mature spawn were unsealed and subsequently transferred to constant-temperature incubators set at 20 ± 0.5 °C, with a relative humidity of 90 ± 3% for a primordia stimulation period lasting four days. Subsequent to the differentiation of primordia, the bags were relocated to fruiting chambers maintained at 25 ± 0.5 °C with a relative humidity of 90 ± 3% and a CO_2_ concentration below 800 ppm. The harvesting process commenced 23 days after the transfer, at which point the cultivated fruiting bodies had achieved morphological equivalence with their wild counterparts and had ceased further growth (Figure 8).

### 3.4. Crude Polysaccharide Extraction and Total Polysaccharide Content Determination

The extraction process yielded 7.07% for cultivated specimens and 6.73% for wild specimens. A glucose standard curve was constructed, resulting in the regression equation y = 0.0069x − 0.125 (R^2^ = 0.9967). Through calculation, the total polysaccharide content was determined to be 24.69% in cultivated specimens and 31.45% in wild specimens.

### 3.5. Antioxidant Activity Assays

#### 3.5.1. Hydroxyl Radical Scavenging Capacity

**Figure 9:**
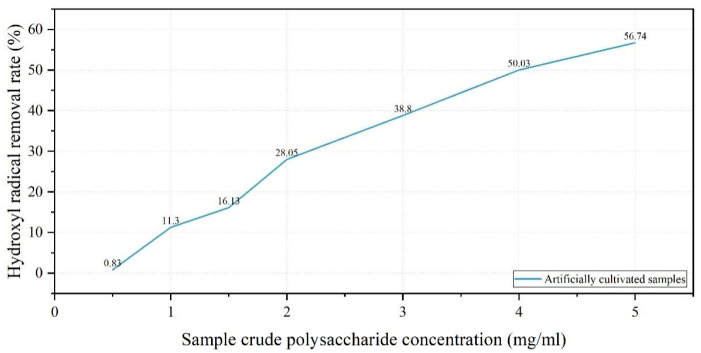
Line graph of hydroxyl radical removal rate.

In this experiment, crude polysaccharide solutions (0.5–5.0 mg/mL) derived from cultivated *Dichomitus squalens* fruiting bodies demonstrated a concentration-dependent positive correlation with hydroxyl radical scavenging activity. The maximum scavenging rate of 56.74% was attained at a polysaccharide concentration of 5.0 mg/mL (Figure 9).

#### 3.5.2. Superoxide Anion Radical Scavenging Capacity

**Figure 10:**
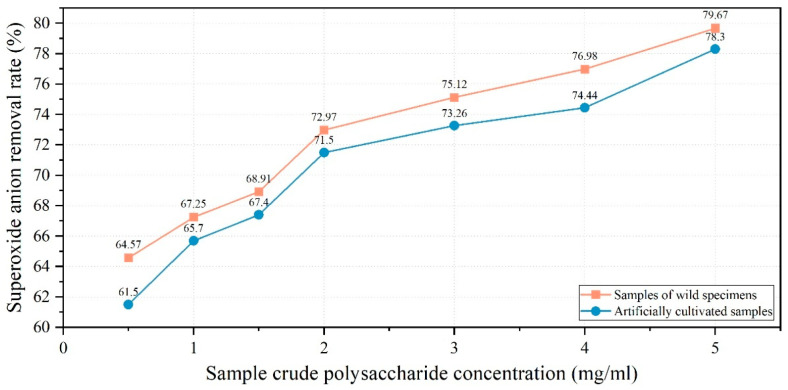
Line graph of superoxide anion removal rate.

The experimental findings indicated that crude polysaccharide solutions (0.5–5.0 mg/mL) derived from both cultivated and wild fruiting bodies of *Dichomitus squalens* demonstrated concentration-dependent positive correlations with superoxide anion radical scavenging capacity. At the highest concentration tested (5.0 mg/mL), polysaccharides derived from cultivated fruiting bodies exhibited a scavenging rate of 78.3%. In contrast, under the same conditions, polysaccharides from wild fruiting bodies demonstrated a slightly higher scavenging efficiency of 79.67% (Figure 10).

## 4. Discussion

In the realm of fungal taxonomy, the microscopic morphological analysis of *Dichomitus squalens* remains underdeveloped. Current identification methods primarily focus on the morphology of hyphae and basidiospores, yet they fall short in thoroughly characterizing ultrastructural features such as basidia, basidioles, and cystidia. The specimens collected from the Xinjiang Uygur Autonomous Region in this study exhibited a high degree of morphological similarity to *D. squalens* specimens from Africa, as reported by Jancovičová [26]. However, subtle differences were noted in the cystidial morphology (17–23 × 4–6 μm, thin-walled, hyaline, irregularly elongate-clavate to fusiform with slightly protruding apices) when compared to the findings of that study.

This study utilized a combined methodology of single-factor and orthogonal experiments to examine the influence of environmental factors—namely carbon source, nitrogen source, pH, and temperature—on hyphal growth while identifying optimal cultivation conditions. The findings revealed that the combination of sucrose and yeast extract powder at pH 5.0 and 30 °C represents the ideal medium conditions for the growth of *Dichomitus squalens*. From a molecular structural standpoint, it is hypothesized that sucrose promotes rapid hyphal growth by decomposing into two monosaccharides, thereby facilitating various efficient metabolic pathways. Although glucose can be directly utilized, its efficacy is limited by a singular metabolic pathway. Conversely, starch, maltose, and dextrin require enzymatic hydrolysis before utilization, leading to a proportional decrease in their growth-promoting effects. The optimal nitrogen source was identified as yeast extract powder, whereas diammonium phosphate and urea were deemed unsuitable, as they reduced hyphal growth rates compared to the nitrogen-free control group. Diammonium phosphate {(NH_4_)_2_HPO_4_} is a compound salt containing ammonium ions (NH_4_^+^) and hydrogen phosphate ions (HPO_4_^2−^). Urea {CO(NH_2_)_2_}, an organic compound comprising a carbonyl group (C=O) and two amino groups (-NH_2_), cannot be directly utilized and requires urease-mediated hydrolysis to release ammonium [33]. However, *D. squalens* lacks this enzymatic capability, resulting in growth inhibition. The overall nitrogen source single-factor experiments demonstrated that inorganic nitrogen sources (ammonium chloride) and simple organic nitrogen sources (peptone) resulted in slower hyphal growth rates compared to yeast extract powder, due to nutritional simplicity or insufficient support. Notably, the hyphal growth rate in the nitrogen-free control group (without exogenous nitrogen addition) was higher than that in the diammonium phosphate group, suggesting that *D. squalens* hyphae possess the ability to store nitrogen compounds. The inhibitory effects of diammonium phosphate and urea on hyphal growth, attributed to toxic effects or metabolic dysfunction, necessitate further investigation into urease activity and phosphorus tolerance to elucidate their metabolic defect mechanisms. In the temperature single-factor experiments, the hyphal growth rate of *D. squalens* exhibited a bell-shaped curve distribution within the 25–35 °C range, with an optimal temperature of 30 °C (16.0292 ± 0.5852 mm/d). Growth rates declined at 35 °C (15.5857 ± 0.9461 mm/d), showing no statistically significant difference from those at 30 °C. However, under orthogonal experimental conditions, the highest hyphal growth rate occurred at 35 °C, suggesting potential experimental variability or interactive effects between factors in the orthogonal design. Wild specimens of *D. squalens* analyzed in this study were collected from Ili Kazakh Autonomous Prefecture, Xinjiang Uygur Autonomous Region, a temperate continental climate zone where the warmest months (July or August) average 20–30 °C [34], aligning with the experimental temperature results. The pH single-factor experiments revealed a negative correlation between hyphal growth rate and pH values in *D. squalens*, with the highest growth rate observed at pH 5.0. *D. squalens* naturally inhabits decaying coniferous woody debris, where the decomposition of resin and tannin during wood decay generates acidic microenvironments [35], aligning with the pH single-factor experimental results. ANOVA of the orthogonal experiment data identified pH as the predominant factor influencing hyphal growth rates.

The entire cultivation process, spanning from the inoculation of the liquid strain to the harvest of the fruiting body, encompassed 57 days, thereby categorizing *Dichomitus squalens* as a slow-growing species within the Polyporaceae family. The growth cycles of related taxa include *Trametes suaveolens* (56 days), *Trametes gibbosa* (57 days), and *Polyporus tuberaster* (45 days) [19,36,37]. The morphological divergence between cultivated and wild fruiting bodies was evident. Cultivated fruiting bodies displayed thinner bases that were closely adherent to the cultivation bags, exhibited upward growth towards the bag openings, and had shorter hemispherical extensions. Additionally, they possessed moist, pure-white pilei that desiccated to a cream-white color, consistent with the characteristics of wild specimens.

Crude polysaccharides were isolated from fruiting bodies utilizing the water extraction and ethanol precipitation technique [23]. The total polysaccharide content was subsequently quantified using the phenol-sulfuric acid method [24]. Experimental findings indicated that cultivated fruiting bodies demonstrated higher crude polysaccharide extraction rates but exhibited lower total polysaccharide content in comparison to their wild counterparts. This discrepancy is likely attributable to: 1. The potential interference from co-extracted proteins necessitates deproteinization purification; 2. Prolonged growth cycles in natural habitats that facilitate sustained polysaccharide accumulation in wild specimens. Assays for hydroxyl radical and superoxide anion radical scavenging revealed a positive correlation between the concentration of *Dichomitus squalens* polysaccharides and their radical scavenging efficiency. At a polysaccharide concentration of 1 mg/mL, the hydroxyl radical scavenging rate (11.3%) was observed to be lower than that of *Inonotus hispidus* (71.5%), *Sparassis latifolia* (54.07%), *Auricularia cornea* (approximately 49%), *Fomitopsis pinicola* (approximately 48%), *Trametes gibbosa* (approximately 38%), *Fomitopsis ostreiformis* (32.61%), and *Panus giganteus* (approximately 26%). The superoxide anion radical scavenging rate was observed to be 65.7%, which is marginally lower than that of *Inonotus hispidus* (69.53%) but exceeds the rates of *Auricularia cornea* (approximately 54%), *Panus giganteus* (approximately 46%), *Trametes gibbosa* (approximately 45%), and *Sparassis latifolia* (15.78%). The data suggest that crude polysaccharides derived from the fruiting bodies of *D. squalens* possess significant superoxide anion radical scavenging activity. Moreover, akin to *Inonotus hispidus*, wild fruiting bodies exhibited superior scavenging rates compared to their cultivated counterparts in superoxide anion radical assays [19,21,38,39,40,41,42].

## 5. Conclusions

*Dichomitus squalens*, a white rot basidiomycete, demonstrates significant potential for various practical applications, including biomedicine, environmental restoration, wastewater treatment optimization, and technological development. This study employed integrated morphological and molecular biological methods to achieve precise identification of *D. squalens*, with optimal cultivation conditions determined as sucrose, yeast extract powder, pH 5.0, and 30 °C, where hyphal growth rate exhibited significant correlation with pH. The complete cultivation cycle—from liquid strain inoculation to fruiting body harvest—required 57 days, yielding cultivated specimens morphologically similar to wild counterparts. The crude polysaccharide extraction rate of cultivated fruiting bodies (7.07%) was slightly higher than that of wild specimens (6.73%), but the total polysaccharide content (24.69%) was lower than that of wild specimens (31.45%), potentially due to shorter accumulation cycles or impurity interference. At 5.0 mg/mL, cultivated polysaccharides achieved hydroxyl radical (OH) and superoxide anion radical (O_2_^−^) scavenging rates of 56.74% and 78.3%, respectively, while wild polysaccharides exhibited higher O_2_^−^ scavenging efficiency (79.67%), demonstrating adaptive antioxidant advantages among related fungi. This study lays the foundation for artificial cultivation of *D. squalens* and exploration of its potential as a natural antioxidant. Future research directions should focus on polysaccharide purification, metabolic mechanism elucidation, and environmental application expansion.

## Figures and Tables

**Table 1:** Names, collection numbers, reported countries, and corresponding GenBank accession numbers of the taxa used in this paper.

Taxon	Collection	Country	GenBank No.	Reference
			ITS	
*Dichomitus albidofuscus*	XSP-1	China	ON127849	
*D*. *albidofuscus*	MUAF843	Czech Republic	EU340897	Tomšovský et al. 2009 [27]
*D*. *albidofuscus*	FCL23	Poland	HQ896245	
*D*. *hubeiensis*	Wei2036	China	JQ780386	Li & Cui 2012 [28]
*D*. *hubeiensis*	Wei2045	China	JQ780387	Li & Cui 2012 [28]
*D. kirkii*	Yuan1237	China	JQ780406	Li & Cui 2012 [28]
*D*. *pendulus*	TL9948	China	JQ780410	Li & Cui 2012 [28]
***D. squalens***	**FJAU66627**	**China**	**PV172452**	**This study**
***D. squalens***	**FJAU66628**	**China**	**PV172453**	**This study**
***D. squalens***	**FJAU66625**	**China**	**PV172454**	**This study**
***D. squalens***	**FJAU66626**	**China**	**PV172455**	**This study**
*D. squalens*	Cui9639	China	JQ780407	Li & Cui 2012 [28]
*D. squalens*	Cui9725	China	JQ780408	Li & Cui 2012 [28]
*D. squalens*	s.n. (H)	Finland	HQ659224	Miettinen & Rajchenberg 2012 [29]
*D. squalens*	LY-AD-421-SS1	USA	KP135330	Floudas & Hibbett 2015 [30]
*D. squalens*		Sweden	JQ518275	Carlsson et al. 2012 [31]
*D. squalens*	Dai15352	China	ON088333	Wang et al. 2022 [32]
*Megasporia sinuosa*	Dai22011	China	ON088321	Wang et al. 2022 [32]
*M*. *sinuosa*	Dai22210	China	ON088322	Wang et al. 2022 [32]

Note: The bolded text in the table pertains to the study species discussed in this article.

**Table 2:** The effects of various single factors on the mycelium growth of wild *Dichomitus squalens*.

Text No.	Factor	Colony Characteristics	Mycelial Growth Rate (mm/d)	Significance	
				0.05	0.01
C1	Glucose	Translucency relatively sparse	11.2 ± 0.61	ab	AB
C2	Sucrose	White relatively sparse	11.72 ± 2.16	a	A
C3	Soluble starch	White relatively sparse	10.5 ± 1.70	b	B
C4	Dextrin	White relatively sparse	10.22 ± 0.66	bc	BC
C5	Maltose	White relatively sparse	10.48 ± 0.67	b	B
C-CK	CK	Translucency relatively sparse	9.30 ± 0.62	c	C
N1	Peptone	White dense	8.59 ± 0.56	c	C
N2	Yeast extract powder	White dense	10.99 ± 0.36	a	A
N3	NH_4_Cl	White dense	9.63 ± 0.74	b	B
N4	Diammonium phosphate	White dense	5.02 ± 0.64	d	D
N5	Carbamide	Not have	0	e	E
N-CK	CK	Transparent sparse	8.99 ± 0.52	c	C
P1	pH 5	White dense	12.52 ± 0.92	a	A
P2	pH 6	White dense	11.20 ± 0.52	b	B
P3	pH 7	White dense	9.50 ± 0.85	c	C
P4	pH 8	White dense	9.34 ± 0.37	c	C
P5	pH 9	White dense	7.77 ± 0.24	d	D
P-CK	Natural conditions	White dense	9.47 ± 0.48	c	C
T1	15 °C	White dense	2.44 ± 0.28	d	D
T2	20 °C	White dense	4.43 ± 0.33	c	C
T3	25 °C	White dense	9.96 ± 0.27	b	B
T4	30 °C	White dense	16.03 ± 0.59	a	A
T5	35 °C	White dense	15.59 ± 0.95	a	A

Note: In the Significance section of the table, letters such as ABCD (or other letters) denote "statistically significant groupings." Groups that share the same letter indicate no significant difference, whereas groups with different letters signify significant differences. Significant differences at the 0.05 and 0.01 levels are represented by lowercase and uppercase letters, respectively.

**Table 3:** Direct-viewing analysis of mycelial growth of *Dichomitus squalens*.

Text No.	Carbon Source	Nitrogen Source	Potential of Hydrogen	Temperature	Mycelial Growth Rate (mm/d)	Mycelial Characteristics
1	1. Glucose	1. Peptone	1. pH5	1. 35 °C	15.40 ± 0.79	White thicker
2	1. Glucose	2. Yeas extract powder	3. pH7	2. 25 °C	6.09 ± 0.29	White dense
3	1. Glucose	3. NH_4_Cl	2. pH6	3. 30 °C	13.17 ± 0.49	White thicker
4	2. Sucrose	1. Peptone	3. pH7	3. 30 °C	7.96 ± 0.54	White thicker
5	2. Sucrose	2. Yeast extract powder	2. pH6	1. 35 °C	16.50 ± 0.42	White dense
6	2. Sucrose	3. NH_4_Cl	1. pH5	2. 25 °C	16.27 ± 1.23	White thicker
7	3. Dextrin	1. Peptone	2. pH6	2. 25 °C	13.27 ± 0.64	White thicker
8	3. Dextrin	2. Yeast extract powder	1. pH5	3. 30 °C	17.83 ± 0.11	White dense
9	3. Dextrin	3. NH_4_Cl	3. pH7	1. 35 °C	3.94 ± 0.85	Translucency sparse
K1	173.27	183.17	247.52	179.17		
K2	203.68	202.1	214.76	178.18		
K3	175.25	166.94	89.92	194.85		
K avg 1	11.55	12.21	16.5	11.94		
K avg 2	13.58	13.47	14.32	11.88		
K avg 3	11.68	11.13	5.99	12.99		
R	2.03	2.34	10.51	1.11		

Note: K value is the sum of mycelial growth rate data at each level of each factor; K average value is the average of mycelial growth rate at each level of each factor; R value is the maximum of K average value minus the minimum of K average value at each factor.

**Table 4:** ANOVA of *Dichomitus squalens*.

Source	Sum of Squares	Df	Mean Square	F	Significance
Carbon source	38.612	2	19.306	42.64	<0.001
Nitrogen source	41.296	2	20.648	45.604	<0.001
Pondus Hydrogenii	922.163	2	461.082	1018.377	<0.001
Temperature	11.662	2	5.831	12.878	<0.001
Error	16.299	36	0.453		
Total	7806.219	45			

## Data Availability

The original contributions presented in this study are included in the article. Further inquiries can be directed to the corresponding author.
